# miR-300 inhibits epithelial to mesenchymal transition and metastasis by targeting Twist in human epithelial cancer

**DOI:** 10.1186/1476-4598-13-121

**Published:** 2014-05-24

**Authors:** Jingshuang Yu, Furong Xie, Xin Bao, Wantao Chen, Qin Xu

**Affiliations:** 1 Department of Oral and Maxillofacial-Head Neck Oncology, Ninth People’s Hospital, Shanghai Jiao Tong University School of Medicine, Shanghai Key Laboratory of Stomatology, Shanghai 200011, China

**Keywords:** microRNA, EMT, Metastasis

## Abstract

**Background:**

Epithelial-to-mesenchymal transition (EMT) is a key step of the progression of tumor cell metastasis. Recent work has demonstrated some miRNAs play critical roles in EMT. In this study, we focused on the roles of miR-300 in regulating EMT.

**Methods:**

The expression levels of miR-300 were examined in epithelial carcinoma cells that underwent an EMT using quantitative reverse transcription-PCR. The role of miR-300 in EMT was investigated by transfection of the miR-300 mimic or inhibitor in natural epithelial-mesenchymal phenotype cell line pairs and in transforming growth factor (TGF) beta-induced EMT cell models. A luciferase reporter assay and a rescue experiment were conducted to confirm the target gene of miR-300. The efficacy of miR-300 against tumor invasion and metastasis was evaluated both *in vitro* and *in vivo*. Correlation analysis between miR-300 expression and the expression levels of its target gene, as well as tumor metastasis was performed in specimens from patients with head and neck squamous cell carcinoma (HNSCC).

**Results:**

MiR-300 was found down-regulated in the HNSCC cells and breast cancer cells that underwent EMT. Ectopic expression of miR-300 effectively blocked TGF-beta-induced EMT and reversed the phenotype of EMT in HN-12 and MDA-MB-231 cells, but inhibition of miR-300 in the epithelial phenotype cells, HN-4 and MCF-7 cells, could induce EMT. The luciferase reporter assay and the rescue assay results showed that miR-300 directly targets the 3′UTR of Twist. Enforced miR-300 expression suppressed cell invasion *in vitro* and experimental metastasis *in vivo*. Clinically, miR-300 expression was found inversely correlated with Twist expression and reduced miR-300 was associated with metastasis in patient specimens.

**Conclusions:**

Down-regulation of miR-300 is required for EMT initiation and maintenance. MiR-300 may negatively regulate EMT by direct targeting Twist and therefore inhibit cancer cell invasion and metastasis, which implicates miR-300 as an attractive candidate for cancer therapy.

## Background

MicroRNAs (miRNAs) are evolutionarily conserved, 20–22 nucleotide noncoding RNAs. They exert their function by binding to the 3′-untranslated region (3′UTR) of a subset of mRNAs resulting in reduced translation of proteins or degradation of the mRNAs [1]. The importance of miRNAs in tumor development and progression has become increasingly evident [2]. Some miRNAs have been shown to exert diverse functions in cancer cell proliferation, angiogenesis and epithelial-to-mesenchymal transition (EMT) [3,4].

EMT is a complex trans-differentiation process in which epithelial cells lose junctional adhesion and adopt a mesenchymal phenotype and morphology. During the EMT process, epithelial cells can migrate through the extracellular matrix. In cancer, EMT allows neoplasic cells to become motile and invasive, and leave the primary epithelial tumor site, thereby contributing to the metastatic potential of carcinomas [5,6].

Transforming growth factor-β (TGF-β) was first described as an inducer of EMT in normal mammary epithelial cells and has since been shown to mediate EMT *in vitro* in most epithelial cells [7,8]. The process of EMT is also regulated by several transcription factors, including Twist (also known as Twist1), SNAIL, SLUG, ZEB1 and ZEB2, as reviewed in [9], which are transcriptional repressors of E-cadherin. Recent work has demonstrated some miRNAs play critical roles in EMT. Members of the miR-200 family are well-established EMT repressors through direct targeting of ZEB1 and ZEB2 [10,11]. However, reports of the roles of other miRNAs in the regulation of EMT are limited.

Recently, using miRNA microarray analysis, we identified a group of differentially expressed miRNAs between mesenchymal-like cancer cells and epithelial-like cancer cells [12]. Remarkably, miR-300 was down-regulated in cancer cells that have undergone EMT comparing with typical epithelial phenotype carcinoma cells, indicating miR-300 might be a regulator of EMT. Based on this finding, miR-300 was chosen for further investigation.

In this study, we verified that miR-300 was down-regulated during an EMT. The low expression of miR-300 plays an important role in EMT-mediated tumor metastasis. Furthermore, we show that Twist is a direct target of miR-300. Ectopic expression of miR-300 could repress invasion *in vitro* and experimental pulmonary metastases *in vivo*. In addition, the expression level of miR-300 is associated with metastatic pattern in oral squamous cell carcinoma.

## Results

### Down-regulation of miR-300 in cells having undergone EMT

**Figure 1:**
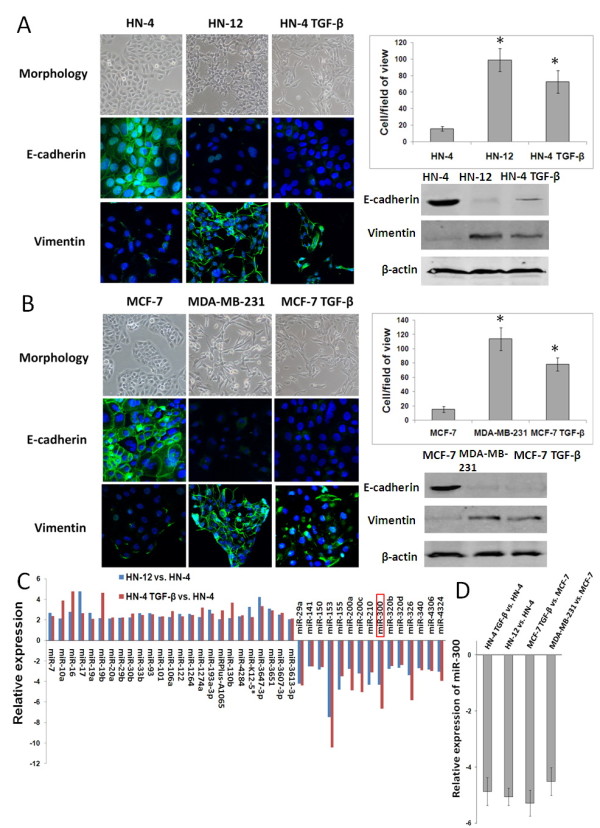
**Down-regulation of miR-300 in cells having undergone EMT. A.,B.** Morphology, E-cadherin and Vimentin expression levels and invasive behavior analysis of HN-4 cells, HN-12 cells and TGF-β-treated HN-4 cells **(A)** or MCF-7 cells, MDA-MB-231 cells and TGF-β-treated MCF-7 cells **(B)**. Left panel showed the phase contrast microscopy and immunofluorescence staining of E-cadherin and Vimentin in the indicated cells. Right-upper panel showed the invasive behavior of the indicated cells in transwell assays. Right-lower panel showed the Western blot analysis of E-cadherin and Vimentin protein levels in the indicated cells. **C**. Fifteen miRNAs, including miR-300, were confirmed down-regulated in the HNSCC cells that underwent EMT by real-time PCR. **D**. The down-regulation of miR-300 was also observed in breast cancer cells that underwent EMT. * *P* < 0.05.

The weakly invasive head and neck squamous cell carcinoma (HNSCC) cell line HN-4 and human breast cancer cell line MCF-7 exhibited a characteristic epithelial cobblestone-like morphology with high expression of the epithelial marker E-cadherin and low expression of the mesenchymal marker Vimentin. In contrast, the highly invasive HNSCC cell line HN-12 and breast cancer cell line MDA-MB-231 were of more elongated, fibroblast-like mesenchymal appearance with reduced E-cadherin and increased Vimentin expression. In addition, HN-4 and MCF-7 cells treated with TGF-β lost their epithelial morphology and acquired mesenchymal traits (Figure 1A,B). These results indicate that the HN-4 and MCF-7 cells are typical epithelial phenotype carcinoma cells, whereas HN-12 and MDA-MB-231 cells have undergone an EMT. Moreover, TGF-β can induce an EMT process in HN-4 and MCF-7 cells. Previously, by miRNA array analysis, we identified a group of differentially expressed miRNAs between epithelial-like cancer cells (HN-4 cells) and mesenchymal-like cancer cells (HN-12 cells and TGF-β induced HN-4 cells) [12]. Expression array data are available at GEO datasets (#GSE38459). In the present study, real-time PCR verification was performed to verify the miRNA array results, which confirmed 15 down-regulated miRNAs in the cells that underwent EMT (Figure 1C). Some of these miRNAs, such as miR-200a and miR-200c, two miR-200 family miRNAs, have been previously demonstrated as EMT negative regulators [13]. In addition to miR-200 family, miR-300 was one of the most down-regulated miRNAs in both HNSCC cells and breast cancer cells that underwent EMT (Figure 1C,D). So far, no direct evidence has been reported that miR-300 may affect EMT, therefore, we selected miR-300 for further analysis as an unknown candidate regulator of EMT.

### Overexpression of miR-300 blocks TGF-β induced EMT

**Figure 2:**
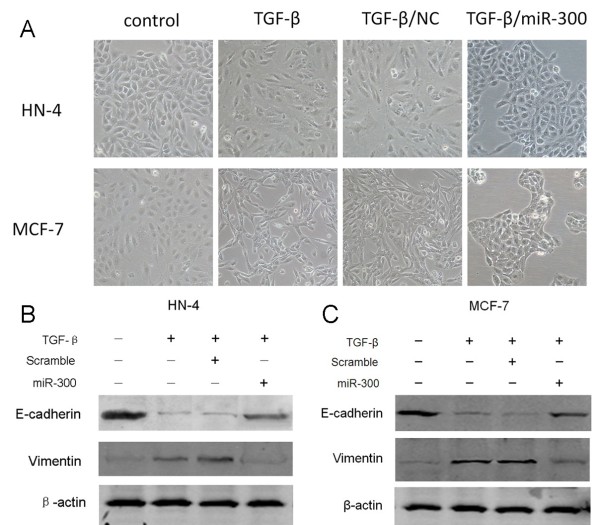
**Overexpression of miR-300 blocks TGF-β induced EMT. A**. Phase contrast microscopy of HN-4 (upper panel) and MCF-7 (lower panel) cells treated with TGF-β alone and co-incubated with miR-300 mimic. **B**. Western blot analysis of E-cadherin and Vimentin staining in HN-4 cells following the mentioned treatments. **C**. Western blot analysis of E-cadherin and Vimentin staining in MCF-7 cells following the mentioned treatments.

Overexpression of miR-300 led to significant resistance to TGF-β induced EMT (Figure 2). The cobblestone-like appearance and E-cadherin expression remained intact and Vimentin expression was attenuated in the cells treated with TGF-β in combination with miR-300. These data suggest that miR-300 is necessary for TGF-β-induced EMT.

### Overexpression of miR-300 reverses the mesenchymal characteristics of HN-12 and MDA-MB-231 cells

**Figure 3:**
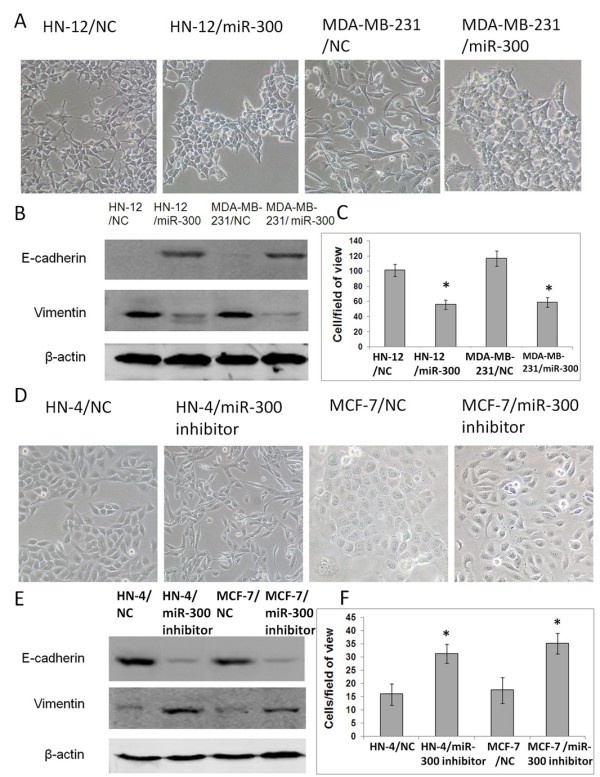
**Down-regulation of miR-300 is required for EMT initiation and maintenance. A**. Ectopic expression of miR-300 produced obvious morphological changes in HN-12 and MDA-MB-231, from spindle-shaped cells to more cobblestone-like cells. **B**. Western Blot analysis revealed a significant increase of E-cadherin expression and reduced Vimentin expression in miR-300 overexpression cells. **C**. Significant decrease of *in vitro* invasive abilities of cells was observed in cells with ectopic expression of miR-300 in transwell assays. **D**. HN-4 and MCF-7 cells with down-regulated level of miR-300 acquired a fibroblastoid appearance. **E**. Western blot confirmed a strong reduction of E-cadherin expression and a concomitant induction of Vimentin in these cells. **F**. Downregulation of miR-300 led to increased cell invasion in transwell assays. * *P* < 0.05.

Next, we tested the ability of miR-300 to reverse the mesenchymal phenotype of highly invasive cancer cells, HN-12 and MDA-MB-231. Firstly, stable cell lines of HN-12 and MDA-MB-231cells with ectopic expression of miR-300 were generated by transfection of miR-300 precursor. Comparing with their parental cells, HN-12 and MDA-MB-231 cells ectopically expressing miR-300 showed an obvious shift in morphology, from spindle-shaped cells to more cobblestone-like cells (Figure 3A). Western Blot analysis revealed a significant increase of E-cadherin expression and reduced Vimentin expression in miR-300 overexpression cells (Figure 3B). Significant decrease of *in vitro* invasive abilities was also observed in these cells (Figure 3C). These results indicate that miR-300 is required for EMT maintenance in mesenchymal phenotype cells.

### Reducing miR-300 level induces EMT in HN-4 and MCF-7 cells

As a consequence of stable knockdown of miR-300, HN-4 and MCF-7 cells showed cellular changes consistent with EMT and increased invasiveness. HN-4 and MCF-7 cells with down-regulated level of miR-300 acquired a fibroblastoid appearance (Figure 3D). Western blot confirmed a strong reduction of E-cadherin expression and a concomitant induction of Vimentin in these cells (Figure 3E). In addition, downregulation of miR-300 led to increased cell invasion (Figure 3F). These results show that reducing endogenous miR-300 expression can efficiently induce EMT.

### Twist is a direct target of miR-300

**Figure 4:**
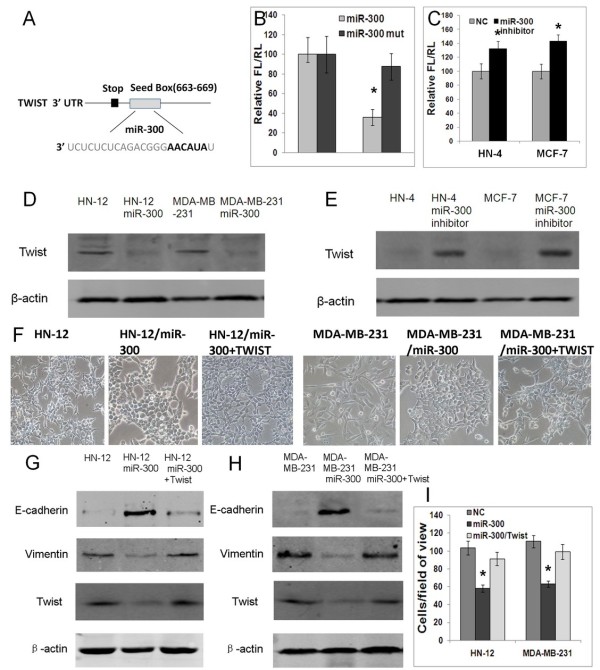
**Twist is a direct target of miR-300. A**. The predicted binding of miR-300 with Twist 3′UTR was illustrated. **B**. A dual-luciferase reporter system analysis was performed to validate miR-300 target genes. A 3′UTR fragment containing the predicted miR-300 targeting sites of Twist was fused downstream of the firefly luciferase gene. A miR-300 mutated binding site (mut) was also constructed. Cotransfection of Twist 3′UTR with miR-300 mimic led to a strong reduction of the luciferase signal. Mutagenesis of the seed region resulted in a reversal of this inhibition. **C**. Endogenous study showed that cotransfection of Twist 3′UTR with miR-300 inhibitor caused an elevated luciferase signal in both HN-4 and MCF-7 cells. **D, E**. Western blot analysis of Twist protein expression in HN-12 and MDA-MB-231 cells treated with miR-153 mimic **(D)** or in HN-4 and MCF-7 cells treated with miR-153 inhibitor **F, G, H, I**. Overexpression of Twist rescued the repressive effects of miR-300 on EMT, leading to morphological changes **(F)**, molecular changes consistent with EMT **(G, H)**, and elevated invasive abilities in transwell assays **(I)**. * *P* < 0.05.

Based on the miR- target analysis using TargetScan website (http://www.targetscan.org), Twist was predicted as a potential target gene of miR-300. The predicted binding of miR-300 with Twist 3′UTR was illustrated (Figure 4A). To examine whether miR-300 directly interacts with the predicted 3′UTR of Twist, the 3′UTR of human Twist was cloned downstream the firefly luciferase coding sequence and co-transfected with miR-300 mimic into 293 T cells. Indeed, normalized firefly luciferase activity decreased by 64% compared with the transfected control. In addition, site-directed mutagenesis of the seed region abolished the inhibitory effect of miR-300 on firefly luciferase activity (Figure 4B). To demonstrate that the endogenous miR-300 can regulate the expression of Twist, the miR-300 inhibitor was transfected into HN-4 and MCF-7 cells. As shown in Figure 4C, the miR-300 inhibitor increased the normalized firefly luciferase activity, as compared to the negative control (NC) (Figure 4C). Twist protein expression decreased when HN-12 and MDA-MB-231 cells were treated with miR-300 mimic and increased when HN-4 and MCF-7 cells were treated with miR-300 inhibitor (Figure 4D,E). To ascertain that the miR-300 regulates EMT through its interaction with Twist, a rescue experiment was also performed. Overexpression of Twist rescued the repressive effects of miR-300 on EMT, leading to morphological and molecular changes consistent with EMT (Figure 4 F,G,H) and elevated invasive abilities in the cells (Figure 4I). These data indicate that miR-300 targets Twist, which in turn results in a negative regulation of EMT.

### miR-300 inhibits the experimental lung metastasis in vivo

**Figure 5:**
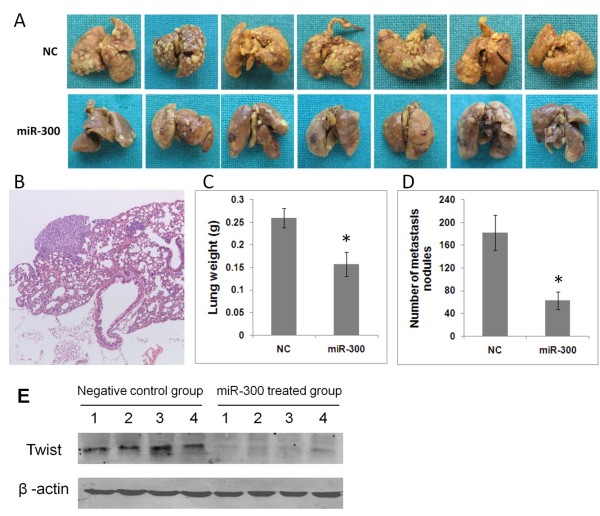
**miR-300 inhibits the experimental lung metastasis *in vivo. *A**. Images of lungs from all mice intravenously injected with MDA-MB-231 cells and with MDA-MB-231cells over-expression of miR-300 (n = 7). **B**. The histological examination of metastatic nodules. **C**. The mean lung weights of the different experimental groups. **D**. The number of tumor nodules on the lung surface of the different experimental groups. **E**. Expression of Twist was analyzed by Western blot in lung homogenates from four randomly selected animals in both groups. * *P* < 0.05.

EMT is a key step of the progression of tumor cell metastasis. Therefore, EMT inhibition may be a way to suppress cancer metastasis [14]. For this purpose, we next investigated whether miR-300 could inhibit the experimental lung metastasis *in vivo.* The lung metastasis models were established by the tail vein injection of MDA-MB-231 cells transfected with a blank vector and MDA-MB-231cells over-expression of miR-300. The metastatic nodules were counted on the surface of lung after 6 weeks and confirmed by histological examination (Figure 5B). As shown in Figure 5A,C,D, the tumor nodules and total lung weight were dramatically reduced in miR-300 over-expression group. The lung weight and the tumor nodules of mice in miR-300 over-expression group were respectively 40% and 66% less than that in negative control group, which indicates miR-300 can inhibit tumor metastasis *in vivo*. Expression of Twist was analyzed by Western blot in lung homogenates from four randomly selected animals in both groups. The expression of Twist was significantly depressed in the miR-300 treated animals compared to negative controls.

### miR-300 expression is inversely correlated with Twist expression and metastasis in tumors from patients with oral cancer

**Figure 6:**
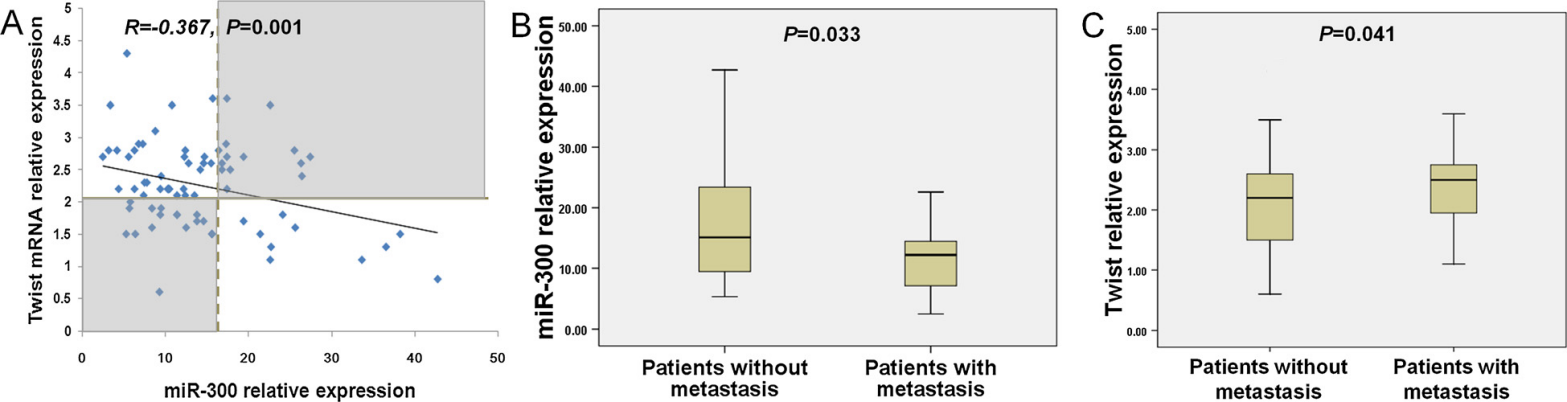
**miR-300 expression is inversely correlated with Twist expression and metastasis in primary tumors from patients with oral cancer. A**. The grey dotted line and the solid grey line indicated the median value of miR-300 and Twist. Expression either above or below the median was considered as “high” and “low,” respectively. Significant inverse correlation was observed between miR-300 and Twist in patient specimens (R = -0.367, *P* = 0.001). White boxes indicated cases with inverse expression pattern of miR-300 and Twist (n = 47), while shaded boxes showed cases with no inverse relationship between miR-300 and Twist (n = 28). **B**. The miR-300 expression level in metastasis-positive patients was significantly lower (*P =*0.033) than that in metastasis-negative patients. **C**. The Twist expression level in metastasis-positive patients was significantly higher (*P =*0.041) than that in metastasis-negative patients.

To address whether the expression of miR-300 is associated with its target, Twist in patients, miR-300 and Twist expression were examined in specimens from 75 patients with oral cancer. It was shown that a significant inverse correlation between Twist and miR-300 expression levels by Pearson correlation analysis (R = -0.367, *P* = 0.001) in these patients (Figure 6A). These data provide further evidence of a functional link between miR-300 and Twist in cancer patients. In addition, the miR-300 expression level in metastasis-positive patients (n = 35) was significantly lower (*P =*0.033) than that in metastasis-negative patients (n = 40) (Figure 6B). Conversely, Twist was expressed at higher levels in patients with metastasis than those without metastasis (*P =*0.041) (Figure 6C).

## Discussion

Metastasis is the primary cause of mortality in most cancer patients [15]. Thus, to understand the molecular mechanisms of metastasis is one of the most important issues in cancer research. Epithelial-mesenchymal transition (EMT), which enables epithelial cells to acquire invasive mesenchymal phenotype, is attracting increasing attention as an important mechanism for the initial step of metastasis [16,17]. Recently, several miRNAs, such as the miR-200 family, have appeared as powerful master regulators of EMT. In this study, we observed that miR-300, a miRNA with so far unknown function in metastasis, played an important role as a suppressor of EMT in human epithelial cancer through direct targeting Twist, which, in turn, inhibited metastasis.

Previously, we identified a group of differentially expressed miRNAs between mesenchymal-like and epithelial-like cancer cells [12]. In this study, these dysregulated miRNAs were verified by real-time PCR in two types of cancer cells, breast cancer cells and oral cancer cells. It was observed that miR-300 was significantly down-regulated both in natural mesenchymal phenotype cells (HN-12 cell and MDA-MB-231 cell) and EMT cell culture models by exposure to TGF-β compared with epithelial phenotype cells (HN-4 cell and MCF-7 cell). These results imply that miR-300 may function as an EMT regulator.

Furthermore, our data demonstrated that over-expression of miR-300 in cancer cells could prevent TGF-β-induced EMT. In addition, the ectopic expression of miR-300 is sufficient to reverse the mesenchymal characteristics and decrease the invasive abilities of mesenchymal phenotype cells. In contrast, by inhibition of miR-300, epithelial phenotype cells could be triggered to undergo EMT, which was accompanied by a more invasive property. Our findings showed that down-regulation of miR-300 is required for EMT initiation and maintenance. More importantly, the involvement of miR-300 in the regulation of EMT was observed in two different types of cancer, breast cancer and oral cancer, suggesting it is a common regulatory mechanism of tumor cell invasion rather than a tissue-specific one.Identifying targets is critical for understanding the biological effects of miRNA [18]. In the present study, Twist was predicted as the target of miR-300 by TargetScan. This interaction between miR-300 and Twist mRNA has not been previously reported. Through dual-luciferase assays, we confirmed the Twist 3′UTR as a direct target of miR-300. The role of Twist in promoting EMT processes has been widely reported [19,20]. Elevated Twist expression was also related with tumor invasion and metastasis in several solid cancers, such as oral cancer [20], esophageal squamous cell carcinomas [21], prostate cancer [22], and uterine cancer [23]. Although Twist has been reported to promote EMT and tumor metastasis through repression of E-cadherin expression, few known miRNA regulators of Twist have been identified to date. In the present study, for the first time, we provided evidence that Twist is a direct target of miR-300. As Twist plays an essential role in EMT as well as tumor metastasis, our data establish a mechanistic link between miR-300, Twist, EMT and tumor metastasis.

Given the critical role of EMT in metastatic tumor formation, inhibition of EMT can be an important therapeutic strategy to inhibit tumor metastasis [24,25]. As a matter of fact, we have shown that miR-300 could decrease *in vitro* invasive behavior of highly invasive cancer cells, suggesting it might be a rational approach of treatment against metastasis. Therefore, we subsequently turned our attention to the anti-metastatic efficacy of miR-300 in a mouse model of experimental lung metastasis. In accordance with our *in vitro* findings, ectopic expression of miR-300 in MDA-MB-231cells led to approximate 2.9-fold decrease in the number of lung metastasis tumor nodules compared with vector control cells, providing *in vivo* evidence that miR-300 has therapeutic potential for metastasis prevention. (HN-12 cells were also employed for animal study, however they failed to develop experimental metastasis model. Data not shown). In addition, levels of Twist protein in lung lysates from miR-300 treated mice were markedly reduced compared with control mice, further confirming that the Twist gene is a true target of miR-300. The miR-300 mediated inhibition of invasion and metastasis likely operates through its suppression effect on EMT.

To gain further insights into the role of miR-300 in oral cancer metastasis, the expression of miR-300 and Twist was detected in 75 oral cancer samples. A significant inverse correlation between miR-300 and Twist expression levels was observed, which confirms the functional interaction of miR-300 and its target Twist. Furthermore, significant lower levels of miR-300 and higher levels of Twist were found in patients with metastasis than that in patients without metastasis. Although low level expression of miR-300 was reported in some types of cancer [26-28], the exact role of miR-300 in cancerous transformation and progression is not clear. Our data provide evidence that down-regulation of miR-300 is involved in metastatic events, which is consistent with the function of miR-300 in modulating EMT. These results reveal a previously unknown function of miR-300 to prevent metastasis by suppressing EMT.

## Conclusions

**Figure 7:**
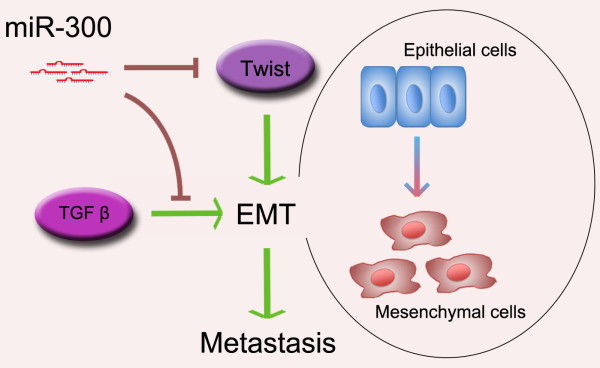
**Schematic diagram of miR-300’s regulation of EMT and metastasis.** By targeting Twist, miR-300 plays an important role in the regulation of EMT.

In summary, our study suggests that down-regulation of miR-300 is required for EMT initiation and maintenance. MiR-300 plays an important role in EMT through direct targeting of Twist. Ectopic expression of miR-300 can reduce invasiveness and metastasis of cancer cells both *in vitro* and *in vivo* (Figure 7).

## Methods

### Cell culture

Two HNSCC cell lines (HN-12 and HN-4 cells) were obtained from the National Institutes of Health (NIH). Two human breast cancer cells (MDA-MB-231and MCF-7 cells) were purchased from American Type Culture Collection (ATCC). All cells were cultured in DMEM containing 10% (v/v) FBS, 100 U/mL of penicillin, and 100 mg/mL of streptomycin at 37°C, 5% CO_2_ in a humidified atmosphere. HN-4 cells are primary squamous cell carcinoma cells of the tongue with low invasive ability, while HN-12 cells were derived from a nodal metastasis in the patient from whom the HN-4 cells originated that have relatively high invasive ability [29]. MDA-MB-231 and MCF-7 cells are commonly used breast cancer cell lines. MCF-7 cells have been found to exhibit low invasive abilities. By contrast, MDA-MB-231 cells have a high invasive potential [30]. Induction of EMT in epithelial cancer cells was initiated by addition of 10 ng/ml TGF-β1 (Invitrogen) to the medium for up to 72 h.

### RNA preparation and quantitative real-time PCR (qRT-PCR)

Total RNA from cultured cells was extracted using Trizol (Invitrogen). Roche’s High Pure miRNA kit was used for total RNA extraction from formalin-fixed paraffin-embedded (FFPE) tissue samples. For quantitative analysis of Twist mRNAs, total RNA was treated with DNase I to eliminate contaminating genomic DNA, and was reverse-transcribed into cDNA with the Reverse Transcriptase MMLV (Takara). The realtime PCR analysis was performed using the SYBR® PrimeScript™ RT-PCR Kit (Takara) on an ABI 7300 Real-Time PCR System. The PCR condition was as follows: 95°C for 5 min, 40 cycles of 30 sec at 95°C, 30 sec at 68°C, 60 seconds at 72°C. All samples were normalized to GAPDH. For quantitative analysis of miRNAs, all the miRNAs in the total RNA samples were polyadenlyated and reverse-transcribed. The resulting cDNA was then subjected to real-time PCR. The entire sequences of miRNAs were used as specific forward primers in combination with the universal reverse qPCR primer provided in the kit. Quantitative Real-time PCR was used under the following thermocycler conditions: 95°C for 30 sec, 40 cycles of 95°C for 5 sec, and 60°C for 30 sec. MiRNA values were normalized to U6 small nuclear RNA from the same sample. The ΔCt and 2^-ΔΔCt^ methods were used for analysis. All the real time PCR reactions were run in triplicate. The specific primer pairs used are listed in the Table 1.

**Table 1:** Primer sequences used in the present study

Gene*	**Primers**
GAPDH F	5′-CATGAGAAGTATGACAACAGCCT-3′
GAPDH R	5′-AGTCCTTCCACGATACCAAAGT-3′
miR-300 F	5′-TATACAAGGGCAGACTCTCTCT-3′
U6 F	5′-CGCAAGGATGACACG CAAATTCGT-3′
Twist 3′UTR F	5′-TCCCCCGGGAAGCAGCTACTGACAGGC-3′
Twist 3′UTR R	5′-CCCAAGCTTGTATGATCTCACCAGGGA-3′
Twist qPCR F	5′-GGACAAGCTGAGCAAGAT-3′
Twist qPCR R	5′-CTCTGGAGGACCTGGTAG-3′

*F, forward; R, reverse.

### Immunofluorescence and confocal laser scanning microscopy

Confocal laser scanning microscopy was performed using a Leica TCS SP2 microscope (Leica Microsystems) as previously reported [31]. Briefly, cells were grown on 10 mm glass coverslips in 24-well culture plates. After methanol fixation for 10 min, cells were incubated with an E-cadherin antibody (1:100, Invitrigen) or Vimentin antibody (1:100, Sigma), followed by incubation with goat-anti-mouse-Alexa 594 conjugated antibodies (1:500, Invitrogen). Cell nuclei were counterstained with DAPI (1:1000, Invitrogen). Confocal image series were recorded on a LEICA TCS SP2 laser scanning microscope.

### Western blot analysis

Cells and murine lung tissue specimens were homogenized in extraction buffer (50 mM HEPES, 250 mM NaCl, 5 mM EDTA, 0.1% NP-40, 1 mM PMSF,1 mM DTT supplemented with Protease inhibitor cocktail). Whole proteins were extracted by centrifugation (12,000 × g) for 10 min at 4°C. Protein concentration was determined using the BCA kit (Pierce). Equal amounts of protein (20 μg/lane) from the cell lysates were electrophoresed under nonreducing conditions on 10% acrylamide gels. After SDS-PAGE, proteins were transferred to a polyvinylidene difluoride membrane. The membrane was incubated for 2 h in PBS plus 0.1% Tween-20 and 5% nonfat skim milk to block nonspecific binding. Subsequently, the membrane was incubated for 2 h with an antibody against E-cadherin (1:500, Invitrigen), Vimentin (1:500, Sigma) and Twist (1:400, Abcam), followed by incubation with fluorescent secondary antibodies (1:5000, IRDye 800 anti-mouse Molecular Probes, Rockland). Blots were stripped and reprobed by using anti-actin antibody (Santa Cruz Biotechnology). Images were acquired with the Odyssey infrared imaging system and analyzed by the software program as specified by Odyssey.

### Transwell invasion assay

Cell invasion was studied using Matrigel-coated transwell inserts as described previously [32]. Briefly, transwell inserts with 8 μm pores were coated with Matrigel (50 μg/well, BD Biosciences). 200 μl of cell suspension (1 × 10^6^/ml) was added to the upper chambers, and 600 μl of DMEM medium containing 5% fetal bovine serum was placed in the lower wells. Thereafter, the cells were incubated for 24 h. Cells that had invaded to lower surface of the Matrigel-coated membrane were fixed with 70% ethanol, stained with hematoxylin, and counted in five random fields under a light microscope.

### Transfection of miRNA mimics and inhibitors

A total of 2 × 10^5^ cells were plated in triplicate overnight in antibiotic-free complete medium in 6-well plates. The cells were then transiently transfected with 200 μL containing 100 nM of a mature miRNA mimic or inhibitor using Lipofectamine 2000 (Invitrogen) according to the manufacturer’s protocol. 72 h after transfection, the total RNA and protein were collected for further analysis.

The sequences of miR-300 were as follows:

Sense: 5′- UAUACAAGGGCAGACUCUCUCU-3′.

Anti-sense: 5′- AGAGAGAGUCUGCCCUUGUAUA-3′.

The sequence of the miR-300 inhibitor was as follows:

5′-GAGAGAGUCUGCCCUUGUAU-3′.

### Generation of stable cell lines with overexpression or knockdown of miR-300

The miR-300 precursor expression plasmid, inhibitor expression plasmid and non-targeting control plasmid were obtained from Genechem. Cells were plated in 6-well plates at a density of 5 × 10^5^ cells per well, and incubated overnight before transfection. Plasmid transfections were performed using Lipofectamine 2000 transfection reagent (Invitrogen) according to the manufacturer’s instructions. After transfection, cells were grown in the presence of Hygromycin (300 μg/ml) to generate stably transfected cell lines.

### Bioinformatic prediction of miR-300 potential targets

The data bases TargetScan (http://www.targetscan.org) was used to predict potential targets for miR-300 [33].

### Luciferase reporter assay

The Twist 3′ UTR segment, which contains the putative binding site for miR-300 was amplified by PCR from HN-12 cells genomic DNA and inserted into the SmaI and HindIII sites downstream of the firefly luciferase coding region of the pGL3 vector (Promega), resulting in pGL3-Twist 3′UTR. Primer sequences are in Table 1. Mutation from GTA to CAT was introduced in the potential miR-300 binding sites (named pGL3-Twist 3′UTR-mut) by using the QuickChange Stratagene method. The correct orientation of the insert was verified by sequencing. 4 × 10^4^ 293 T cells were cultured in 24-well plates and cotransfected with 0.15 μg of either pGL3-Twist 3′UTR or pGL3-Twist 3′UTR-mut together with 0.05 μg of the control vector containing renilla luciferase, pRL-TK (Promega) and 40 pmol of miR-300 mimics. Transfection was done using Lipofectamine 2000. Twenty-four hours after transfection, firefly and renilla luciferase activity were measured using the Dual-Luciferase Reporter Assay (Promega) and firefly luciferase activity was normalized to renilla luciferase activity. Each transfection was repeated twice in triplicate. To assess the effect of the endogenous miR-300, luciferase activities were also measured in HN-4 and MCF-7 cells in the presence of miR-300 inhibitor.

### Rescue experiment

To further validate direct targeting of Twist by miR-300, functional rescue experiment was performed by co-transfection with miR-300 mimic and plasmid constructs expressing Twist in HN-12 cells and MDA-MB-231 cells using Lipofectamine 2000 as described above. The expression plasmid pcDNA-Twist, encoding human Twist was purchased from Genechem. The presence of complete Twist coding regions was confirmed by DNA sequencing.

### Experimental metastasis

All animal studies were conducted in accordance with an Institutional Animal Care and Use Committee-approved protocol at the Shanghai Jiao Tong University. 5-week-old female nude BALB/c mice were used in the study. MDA-MB-231 cells stably transfected with miR-300 or with negative control vector (2 × 10^6^ cells in 0.2 ml PBS) were injected into mice via the tail vein. Each treatment group consisted of 7 mice. After 6 weeks, the mice were necropsied, and the lungs were removed. Visible lung metastases were counted in Bouin’s fixed tissues with the aid of a dissecting microscope [34].

### Clinical sample

A total of seventy-five FFPE tissue specimens were obtained from patients who were diagnosed with oral squamous cell carcinoma (OSCC) and underwent surgical resection between 2006 and 2007 in the Department of Oral Pathology, Ninth People’s Hospital, Shanghai Jiao Tong University School of Medicine, with (n = 35) or without metastasis (n = 40). Real-time PCR analysis for miR-300 and Twist mRNA was performed as described above. Primer sequences are in Table 1. Correlation analysis between miR-300 level and Twist mRNA expression was performed. The median value was used as cutoff point for separating miR-300 high and low expression cases.

### Statistical analysis

All data are presented as the mean value ± SD. Differences between individual groups were analyzed by t-tests. *P* values less than 0.05 (two-sided) were considered statistically significant. In order to determine the correlation coefficient (R) and the significance (*P*) between the expression of miR-300 and Twist mRNA, the Pearson’s correlation algorithm was applied. Statistical analyses were performed using the SPSS 11.5 software (SPSS Inc).

## Competing interests

The authors declare that they have no competing interests.

## Authors’ contributions

WC and QX designed this research work. JY and FX performed experiments and analyzed data in this manuscript. JY and QX interpreted the data. JY, FX and XB wrote the manuscript. All authors read and approved the final manuscript.
